# Endotracheal intubation skills of pediatricians versus anesthetists in neonates and children

**DOI:** 10.1007/s00431-019-03395-8

**Published:** 2019-06-08

**Authors:** Sam J. van Sambeeck, Sander M. J. van Kuijk, Boris W. Kramer, Petronella M. Vermeulen, Gijs D. Vos

**Affiliations:** 1 Department of Pediatrics, Maastricht University Medical Centre, P. Debyelaan 25, P.O. Box 5800, 6202 AZ Maastricht, The Netherlands; 2 Department of Clinical Epidemiology and Medical Technology Assessment, Maastricht University Medical Centre, P. Debyelaan 25, P.O. Box 5800, 6202 AZ Maastricht, The Netherlands; 3 Department of Anesthesiology, Maastricht University Medical Centre, P. Debyelaan 25, P.O. Box 5800, 6202 AZ Maastricht, The Netherlands

**Keywords:** Child, Infant, Newborn, Endotracheal intubation, General hospitals

## Abstract

This study compares the performance of pediatricians and anesthetists in neonatal and pediatric endotracheal intubations (ETI) during simulated settings. Participants completed a questionnaire and performed an ETI scenario on a neonatal and a child manikin. The procedures were recorded with head cameras and cameras attached to standard laryngoscope blades. The outcomes were successful intubation, time to successful intubation, number of attempts, complications, total performance score, end-assessment rating, and an assessment whether the participant was sufficiently able to perform an ETI. Fifty-two pediatricians and 52 anesthetists were included. For the neonatal ETI, the rate of successful intubation was in favor of anesthetists although not significant. Anesthetists performed significantly better in all other outcomes. Of the pediatricians, 65% was rated sufficiently adept to perform a neonatal ETI vs 100% of the anesthetists. Pediatricians (29%) overestimated while anesthetists (33%) underestimated their performance in neonatal ETI. For the pediatric ETI, all outcomes were significantly better for anesthetists. Only 15% of all pediatricians were considered sufficiently able to perform pediatric ETI vs 94% of the anesthetists.

*Conclusion*: Anesthetists are far more adept in performing ETI in neonates and children compared with pediatricians in a simulated setting. Complications are expected to occur less frequently and less seriously when anesthetists perform ETI.

**Table:** 

**What is Known:** • *Endotracheal intubation (ETI) performed by inexperienced care providers can lead to unsuccessful and/or prolonged intubation attempts. This can cause complications such as hypoxemia, trauma to the oropharynx and larynx, and prolonged interruption of resuscitation, which results in a high morbidity/mortality.* • *Fifty to 60 real-life ETI procedures are needed before ETI can be performed with a 90% success rate. Despite this, 18% of providers still require some assistance even after performing 80 intubations. Skill fade will occur if there is too little exposure.*	
**What is New:** • *This study shows that, on both neonatal and child manikins, anesthetists perform better in ETI compared with pediatricians. Besides this, complications are expected to occur less frequently and less seriously when anesthetists are performing the ETIs on neonates and children.* • *In those countries where there are no clear interprofessional agreements made in general hospitals on who will perform ETI on neonates and children in acute care settings, these agreements are urgently necessary.*

**Electronic supplementary material:**

The online version of this article (10.1007/s00431-019-03395-8) contains supplementary material, which is available to authorized users.

## Introduction

Endotracheal intubation (ETI) is the golden standard for securing the airway in situations where the provider is unable to ventilate the patient adequately with a bag-and-mask or by a supraglottic airway device, or if an open airway is compromised [20, 24]. Unsuccessful intubation attempts lead to complications, resulting in a high morbidity/mortality rate [1, 2, 18, 21]. There is a direct correlation between the experience of the ETI provider and the success rate, intubation time, and number of attempts needed for each ETI [3, 9, 13, 22, 25]. In Dutch general hospitals, the attending pediatrician is responsible for the acute care of critically ill neonates and children, sometimes in co-operation with emergency physicians, anesthetists, intensivists, and nursing staff [14]. In Dutch general hospitals, there is no pediatric intensive care specialist available who can perform pediatric ETI in acute settings. In these hospitals, due to the low incidence of critically ill children and newborns requiring an acute ETI [7, 11, 17], the pediatricians’ exposure to ETI is expected to be low [14, 17]. Our hypothesis is that, since anesthetists have more experience in ETI, they have better intubation skills and higher success rates than pediatricians in ETIs. The study objectives are (1) to explore the actual exposure of general pediatricians and anesthetists to ETI in neonates and children; (2) to compare the intubation skills (success rate, intubation time, number of attempts, degree of laryngeal view, complications, and overall performance) of both groups in a neonatal and a child manikin setting; and (3) to compare the self-perceived capability of the ETI performance with the actual performance on the manikins.

## Materials and methods

### Study design

A cross-sectional study was performed among pediatricians and anesthetists, practicing in general hospitals. At their respective annual national medical conferences, specialists were asked to volunteer to perform ETI procedures on a neonate and a child manikin. Exclusion criteria for participants were (1) working in a tertiary facility or university hospital, (2) not participating in neonatal care or acute care of critically ill children, and (3) not participating in on-call duties.

The research and scenario setup were standardized for all settings. All participants started with an electronic survey (Appendix A), after which they continued with the intubation scenarios.

### Procedures

The electronic survey consisted of a general section concerning age and time since completion of residency, a second section concerning exposure to ETI in neonates and children in the past year, and their self-perceived competence. Also, participants were asked their opinion about which medical specialist would be the most suited to perform an ETI on neonates and children. General questions were multiple choices. Questions concerning their self-perceived competence or opinions were based on a five-point Likert scale (1 = completely incompetent to 5 = highly competent) and (1 = not at all preferred to 5 = very preferred). The questionnaires were anonymous, coded per specialty, and linked to the intubation performances on both manikins.

After completing the survey, the participant was equipped with a head camera (Go Pro Hero4 Silver®, San Mateo, USA) and proceeded to the intubation scenario on the neonatal and child manikin. The setup of both manikins was identical, apart from size. Participants received information about the manikins’ age and clinical condition. They were asked to perform an ETI by direct laryngoscopy on the neonatal and child manikins, just as they would perform it in real-life situations. The intubation procedures were filmed in overview by the head camera. The view from the laryngoscope blades was filmed with 5-mm cameras with lighting (Waterproof Endoscope Camera® USB 5 mm 6LED, J&S United Technology, Taipei, Taiwan), attached to standard laryngoscope blades (Macintosh and Miller), replacing the original light source. The laryngoscope blades and corresponding handles were similar to those used in daily clinical practice. The two manikin scenarios were (1) neonatal manikin (Newborn Anne, Laerdal Medical®, Stavanger, Norway), representing a full-term newborn female, weight 3500 g. She was born in the delivery room after an uncomplicated pregnancy. At birth, there was no spontaneous breathing after five sustained insufflation breaths. The circulation was normal. (2) Child manikin (SimJunior, Laerdal Medical®, Stavanger, Norway), representing a 6-year-old previously healthy boy admitted to the emergency department with acute respiratory insufficiency and secondary apnea, but with normal circulation. The manikin was placed in supine face straight-up position, the table height was 78 cm. To perform the intubation, participants could use different laryngoscope blades (Miller size 1 and Macintosh size 1–4), endotracheal tubes (sizes 2.5–6.5, cuffed and uncuffed), Magill forceps (size 7 child and 9 adult), and stylet. Bag-valve-mask resuscitator (500 ml and 1600 ml) and masks (sizes 0–5) were available to perform bag-mask ventilation (Laerdal Silicone Resuscitator, Laerdal Medical®, Stavanger, Norway). There was one assistant available per station, who could reach for materials when asked for. There was no feedback of vital signs or patient status given during the procedure.

### Outcomes

Three experts, blinded for the specialty of the participants, individually rated all the videos of the performances of the participants in different orders. These experts, further referred to as observers, consisted of a senior pediatric-intensivist, a senior neonatologist, and a senior pediatric-anesthetist, all working in a tertiary facility university medical center and with extensive experience in airway management. All observers rated the intubation procedures of the participants independently, using the footage from the overview head camera and the laryngoscope blade cameras. A predefined 8-item scoring list (Appendix B) was used to rate the intubation performance, further referred to as the total performance score. Higher scores indicate better performance; positive and negative ratings could be given to different components, with a maximum total score of 13 points. This total performance score was based on (1) steps outlined in the Advanced Pediatric Life Support (APLS) and European Pediatric Advanced Life Support (EPALS) airway management checklists and (2) expert opinion by the observers.

The primary outcome of this study was the intubation success rate defined as an endotracheal tube placed through the vocal cords. Secondary outcomes included the time to successful intubation, the number of attempts, degree of laryngeal view, the number of complications (laryngoscope blade between the vocal cords, esophagus intubation, transferring the laryngoscope handle from one hand to the other during intubation and incorrect cuff placement (between the vocal cords)), and the total performance score (see Appendix B). In addition, an end-assessment rating from 1 to 10 (1 being the lowest and 10 being the highest score) and the impression whether the participant was sufficiently capable to perform a safe ETI in a neonate and a child were secondary outcomes.

### Statistical analysis

Characteristics of the participants were reported as absolute values and percentages, stratified by specialty. Differences between pediatricians and anesthetists were tested using Pearson’s chi-squared test.

For both manikins, the outcome measures of the total performance score and the end-assessment grade between pediatricians and anesthetists were tested using the Mann-Whitney *U* test. Differences in the proportion of participants that performed successful ETI and the proportion of participants that were found sufficiently able to perform the procedures were tested using Pearson’s chi-squared statistic. In case of expected cell counts of five or less, we used Fisher’s exact test. The difference between pediatricians and anesthetists on the number of attempts needed for successful ETI was tested using the non-parametric Mann-Whitney *U* test, while the difference in total time required to perform ETI was tested using the independent *t* test.

In case of disagreement between observers on categorical scales, the category that was scored by the majority was used for the analysis. Otherwise, the average score was used for the analysis for continuous items. We used Cohen’s kappa to determine agreement between observers for binary items, and the intra-class correlation coefficient (ICC) for (semi-) continuous variables. In case of perfect agreement on a binary item, only the total agreement was computed. Self-reported clinical experience and the self-perceived capability of the participants are tested for differences between pediatricians and anesthetists using Pearson’s chi-squared test. All analyses were performed using IBM (New York, USA) SPSS version 23. Figures were made in R version 3.3.3 (Vienna, Austria).

## Results

Out of 132 participants, 104 were eligible for analysis. Twenty-eight (21.1%) participants were excluded because of incomplete survey, incorrect instructions, no on-call duty, or video/camera error (e.g., incomplete view). Out of these 104 participants, 52 were registered pediatricians and 52 registered anesthetists.

### Electronic survey

Characteristics of the participating physicians are shown in Table 1. There were no statistically significant differences in age or length of time since completion of residency between both groups. Questionnaire responses of the participants about existing agreements on who is performing ETI in neonatal and pediatric acute care settings and who preferably should perform ETI are shown in Table 1 as well. An online supplement (Table 5a and 5b) shows the pre-study ETI training of the participant in the past year.

**Table 1:** Characteristics and questionnaire responses of all participants, stratified by specialty

	Pediatricians (*n* = 52)	Anesthetists (*n* = 52)	*p* value for difference
Age			0.138
< 40 years	8 (15.4%)	10 (19.2%)	
40–50 years	32 (61.5%)	21 (40.4%)	
51–60 years	7 (13.5%)	15 (28.8%)	
> 60 years	5 (9.6%)	6 (11.5%)	
Time since completion of residency			0.162
< 5 years	6 (11.5%)	9 (17.3%)	
5–10 years	11 (21.2%)	8 (15.4%)	
11–20 years	27 (51.9%)	19 (36.5%)	
> 20 years	8 (15.4%)	16 (30.8%)	
Are there written agreements about who performs ETI in neonates and children?			0.066
Yes	11 (21.2%)	22 (42.3%)	
No	15 (28.8%)	10 (19.2%)	
Do not know	26 (50.0%)	20 (38.5%)	
Who is performing ETI in neonates and children?			0.047
Pediatrician	2 (3.8%)	11 (21.2%)	
Anesthetist	29 (55.8%)	21 (40.4%)	
Pediatrician in neonates, anesthetist in children	5 (9.6%)	2 (3.8%)	
Do not know	12 (23.1%)	16 (30.8%)	
Otherwise (“most capable person”)	4 (7.7%)	2 (3.8%)	
Is it preferred that anesthetist perform the neonatal ETI?			0.010
Not preferred	22 (42.3%)	7 (13.5%)	
Neutral	21 (40.4%)	31 (59.6%)	
Preferred	9 (17.3%)	14 (26.9%)	
Is it preferred that anesthetist perform the pediatric ETI?			0.030
Not preferred	4 (7.7%)	5 (9.6%)	
Neutral	9 (17.3%)	23 (44.2%)	
Preferred	39 (75.0%)	24 (46.1%)	

**Fig. 1:**
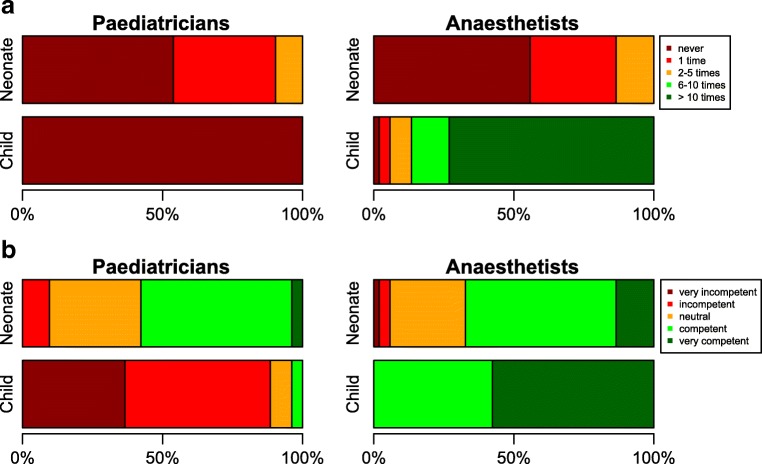
**a** Self-reported experiences (on a yearly basis) with endotracheal intubation in neonates and children. **b** Self-perceived capability of performing endotracheal intubation on neonates and children

Figure 1a shows the distribution of self-reported experiences with ETI of both groups’ participants over the past year. On average, anesthetists reported to have performed pediatric ETI more often than pediatricians, not statistically significant for ETI in neonates (*p* = 0.738), but statistically significant for ETI in children (*p* < 0.001). Figure 1b shows the distribution of self-perceived capability of performing ETI on neonates and children. The self-perceived capability of performing ETI in children was statistically significantly higher in anesthetists compared with pediatricians (*p* < 0.001).

### Endo-tracheal intubation performance

#### Neonatal ETI

We observed a non-significant difference in the proportion of pediatricians versus anesthetists that performed a successful neonatal ETI (i.e., tube in larynx through vocal cords) (90.4% versus 100%, *p* = 0.057). On all other scores, there was a significant difference in performance all in favor of the anesthetists.

**Fig. 2:**
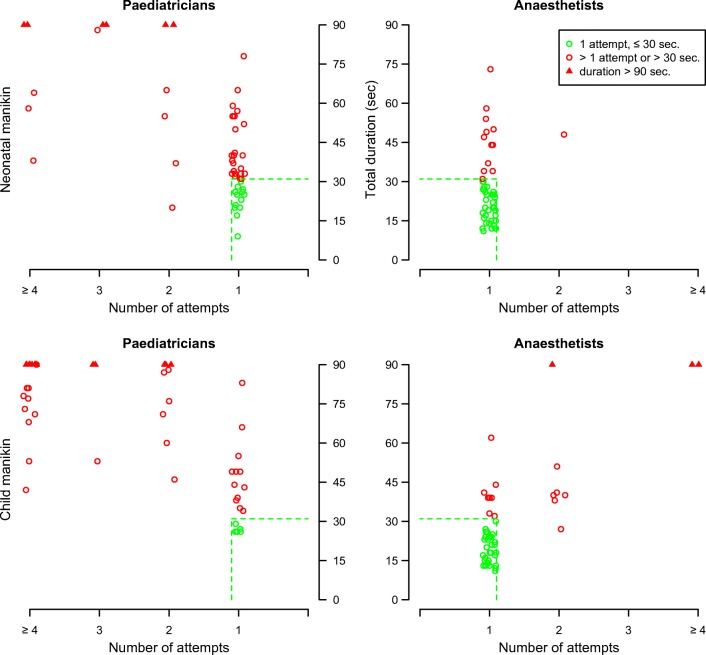
Distribution of the time and number of attempts needed to perform ETI on both manikins per specialty (mirror wise)

**Fig. 3:**
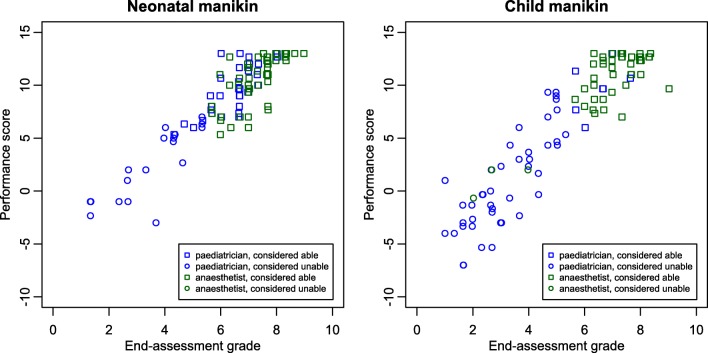
Distribution of the end-assessment grade, the total performance score, and the consideration (in)sufficiently able to perform ETI on both manikins per specialty

Thirty-eight (73.1%) pediatricians succeeded the ETI in one attempt, compared with 51 (98.1%) of the anesthetists (*p* = 0.001). Figure 2 (upper half) shows the distribution of the time and number of attempts needed to perform an ETI per specialty. On average, pediatricians needed 47.7 s to perform ETI compared with 27.1 s by the anesthetists (*p* < 0.001). The median total performance score using the predefined scoring list (Appendix B) was 7.5 for the pediatricians compared with 11.5 for the anesthetists (*p* < 0.001). The median number of complications was 1.0 for the pediatricians versus zero for the anesthetists (*p* ≤ 0.001). Pediatricians scored a significantly lower end-assessment grade compared with the anesthetists (median 5.8 versus 7.6, *p* < 0.001). For the neonatal manikin, 34 (65.4%) pediatricians were considered sufficiently able to perform the procedure, compared with 52 (100%) of the anesthetists (*p* < 0.001). Figure 3 shows the distribution of the end-assessment grade, the total performance score, and the consideration “(in)sufficiently able to perform ETI” per specialty.

#### Pediatric ETI

The difference in a successful pediatric ETI was statistically significant (pediatricians 57.7% versus anesthetists 96.2%, *p* < 0.001). Eighteen (34.6%) pediatricians succeeded in one attempt versus 43 (82.7%) of the anesthetists (*p* < 0.001). Two pediatricians (4%) refused to perform the procedure on the child manikin, since they felt not capable of performing the procedure and they would not perform this procedure in their hospital.

Figure 2 (lower half) shows the distribution of the time and number of attempts needed to perform a pediatric ETI per specialty. Pediatricians needed 83.4 s to perform ETI compared with 33.6 s by the anesthetists (*p* < 0.001). The median total performance score was 2 for the pediatricians versus 12.3 for the anesthetists (*p* < 0.001). The median number of complications (see also Table 2) was 2.0 for the pediatricians versus zero for the anesthetists (*p* < 0.001). Pediatricians scored a significantly lower end-assessment grade for the total procedure with a median score of 3.5 versus 7.3 for the anesthetists (*p* < 0.001).

**Table 2:** ETI complications on the neonatal and child manikin stratified by specialty

		Pediatricians (*n*)	Anesthetists (*n*)	*p* value
Blade between vocal cords	Neonate Child	16 (30.8%) 25 (48.1%)	9 (17.3%) 8 (15.4%)	0.108 < 0.001
Tube in esophagus	Neonate Child	8 (15.4%) 21 (42.0%)	0 (0.0%) 3 (12.5%)	0.060 < 0.001
Transferring laryngoscope (form one hand to the other)	Neonate Child	16 (30.8%) 19 (38.0%)	1 (1.9%) 2 (3.8%)	< 0.001 < 0.001
Incorrect cuff placement (cuff between vocal cords)	Neonate	-	-	-
	Child	3 (9.7%)	0 (0.0%)	0.114

For the child manikin, 8 (15.4%) pediatricians were considered sufficiently able to perform the procedure, compared with 49 (94.2%) of the anesthetists (*p* < 0.001).

Table 3 shows the distribution of self-perceived capability of performing ETI in comparison with the qualification given by the observers: in some participants, there is a discrepancy between their self-perceived capability and the assessed performance.

**Table 3:** Self-perceived capability of performing ETI on neonates and children by pediatricians versus anesthetists, in comparison with their performance on a neonatal and child manikin

	Neonate		Child	
	Pediatricians	Anesthetists	Pediatricians	Anesthetists
Aware of competency	42.3%	67.3%	0.0%	94.5%
Not aware of competency	23.1%	32.7%	15.7%	0.0%
Aware of lack of competency	5.8%	0.0%	74.5%	0.0%
Unaware of lack of competency	28.8%	0.0%	9.8%	5.8%

#### Intra-class correlation coefficient

For the median total performance score, the observers had a very high agreement (ICC = 0.983 for the neonate, 0.989 for the child). There was complete agreement between the three observers for procedures on both manikins with respect to the complications (ICC = 1.00). The observers’ ICC was 0.81 and 0.86 respectively for the end-assessment grades of the participants on the neonatal and child manikin. The observers had 100% agreement on the ratings whether the participant was considered sufficiently able to perform the procedure.

## Discussion

This study shows that anesthetists are better in performing ETIs on both neonatal and child manikin and perform significantly better on most components compared with pediatricians, resulting in a higher success rate and fewer complications. The main differences occurred at the stage of ETI, such as laryngoscopy and advancing the tube into the glottis and trachea. Although most of us would expect these findings, no study has ever been published, as far as we know, that compared the intubation skills of pediatricians with those of anesthesiologists in a standardized setting. Although there was no significant difference between both groups of specialists in successful neonatal ETI (i.e., tube in larynx through vocal cords), the performance of the total procedure by the anesthetists was significantly better on all other components, expecting fewer complications.

The majority of the Dutch pediatric and anesthetic participants still believed that pediatricians should perform the emergent neonatal intubation: most probably based on a historical basis. Yet, the anesthetist appears to be the most qualified person to carry out this procedure in general hospitals. Studies have shown that 50–60 ETI real-life procedures need to be conducted to achieve a 90% success rate in controlled settings [13, 22, 25]. However, 18% of providers still require assistance after 80 intubations [13]. These numbers will never be achieved by pediatricians in (Dutch) general hospitals. The exposure of pediatric residents to ETI is very low as well. In the Netherlands, neonatal and pediatric ETI is not an obligatory “entrustable professional activity” anymore during residency training [5, 10, 23]. Studies on neonatal ETI by pediatric residents show a low success rate of 20–26% [6, 12, 15, 19]. This lack of exposure to ETI cannot simply be replaced by a simulation-based manikin training, since this training is not a guarantee for successful skills in the acute care setting [4, 8].

Although Dutch general anesthetists also have limited exposure in performing neonatal ETI in particular, in general, they are far more expert in performing ETI procedures. They are highly skilled in airway management including the avoidance of potential serious complications. This makes them the most suitable persons to perform ETI in neonates and children in acute care settings.

There are countries, like the UK, that have made a national agreement that the anesthetist performs ETI in all neonates and children that require an emergent ETI. In our opinion, there is an urgent need to make this agreement in those countries that did not make this agreement yet. Our finding that quite a large number of the anesthetists is not aware of their own competence in performing neonatal ETI and one quarter of the pediatricians rated themselves capable to perform neonatal ETI while they were not skilled enough makes it unlikely that both occupational groups can make these agreements at hospital level. The national societies of anesthesia and pediatrics must take the lead and the responsibility to come to national agreements and directives.

### Facilitation of change

Although the anesthetist is the most skilled person to perform the emergent ETI, the implementation of new national agreements on this subject might not be easy. Anesthetists have a low self-perceived competence in performing neonatal ETI and according to the results in the survey, a minority of the anesthetists stated that they should be responsible for the ETI in neonates. The restraint of some anesthetists to perform an emergency neonatal ETI is probably due to the difficulty to maintain neonatal ETI skills in general hospitals since there is a lack of regular on-site ETIs in neonates.

To address this problem, (1) an extensive, advanced pediatric airway management training course should be developed at a national level for general anesthetists/residents in anesthesia, and be mandatory both during residency and for re-registration. To maintain adequate skill, attendance at the national courses and local skill training programs and assessments are needed since, over time, there is significant decay in skills when not frequently used or refreshed [16, 26], especially in the field of neonatal intubation. (2) Tertiary care centers do have to play a key role in facilitating regional training facilities to enable anesthetists from general hospitals to remain skilled in and confident on neonatal ETI. (3) It is of no use to train general pediatricians in performing ETI since they cannot gain the practical airway-management experience needed to adequately perform ETI. Instead, there should be developed a national course for pediatricians/residents in pediatrics to obtain and maintain the skills in non-invasive maneuvers to guarantee a free airway, mask-and-bag ventilation and the introduction of a supraglottic airway device. Also, this course should be mandatory during residency and for re-registration in pediatrics.

### Limitations

Literature shows that skills learned on manikins are not absolute guarantee for success in real-life situations [4, 8]. However, for this study, we had to use manikins, since it was impossible to perform the meticulous study in vivo on neonates and children. This was the best study design we could conceive to compare the two groups of medical specialists in a precise standardized setting. The restrictions given by the manikins were the same for both groups. Our study fulfills partially Kane’s validity framework concerning the assessment strategy that was used to assess the ETI performance. Concerning the data evaluating “scoring,” we tried to achieve a form of inter-item correlation by comparing the objective score (total performance score) with the subjective scores (end-assessment rating and an assessment) of the observers, to see if high total performance scores did correlate with high subjective scores and vice versa, which was the case. Besides this, we tested the inter-rater reliability, which was strong. With respect to the “generalizability,” the overall inter-station reliability was high since the test stations were similar during all test situations. The materials used were identical during all tests and identical to the materials used in daily practice. The same introduction and instructions were given to the participant by the same instructors in all test situations. The data evaluating “extrapolation and implications” could not be executed. We did not correlate assessment scores by clinical errors or failure in practice since the participants were not tested in practice. In summary, we suggest the evidence for scoring and generalization supports the use of our simulation-based assessment strategy as a reflection of ETI performance in a simulated setting. However, in the extrapolation to daily practice, evidence is lacking, since test scores were not correlated to real clinical performances—mainly due to lack of clinical exposure by the participants. However, performing ETI on manikins is expected to be easier than performing ETI in practice, in particular in acute care settings. We therefore hypothesize that low assessment scores in the manikin model reflect low real-life ETI performance.

## Conclusions

With this study, we determined that anesthetists are more successful and better qualified in intubating neonates and children compared with pediatricians in a simulated setting. Clear agreements between these specialties need to be made urgently and translated in concrete national and local protocols with the main appointment that, in general, the anesthetist will perform ETI on neonates and children in acute care settings.

## Electronic supplementary material

ESM 1 (DOCX 19 kb)

ESM 2 (DOCX 101 kb)

## Abbreviations

APLS: Advanced Pediatric Life Support
EPALS: European Pediatric Advanced Life Support
ETI: Endo-tracheal intubation
NLS: Neonatal Life Support
