# Supplementary material for: Endotracheal intubation skills of pediatricians versus anesthetists in neonates and children
Source: Eur J Pediatr. 2019 Jun 8;178(8):1219–27. doi: 10.1007/s00431-019-03395-8 (PMC6647518; doi:10.1007/s00431-019-03395-8)
Supplement: Supplementary file 1 — (DOCX 19 kb) [file 431_2019_3395_MOESM1_ESM.docx]

**ONLINE SUPPLEMENT**

**Table 4a** Frequency of bag-mask or Neopuff ventilation in the past year per age category and per specialty in percentages.

*Ped = Pediatricians, Ane = Anesthetists. Neonate 0-4 weeks, infant 4 weeks-1 year, child 1-16 years.*

|  | **Neonate** | | **Infant** | | **Child** | |
| --- | --- | --- | --- | --- | --- | --- |
|  | **Ped** | **Ane** | **Ped** | **Ane** | **Ped** | **Ane** |
| **Never**  **1 time**  **2-5 times**  **6-10 times**  **>10 times** | 3.8  7.7  36.5  32.7  19.2 | 34.6  36.5  25.0  1.9  1.9 | 55.8  25.0  15.4  0.0  3.8 | 13.5  19.2  40.4  7.7  19.2 | 65.4  23.1  11.5  0.0  0.0 | 1.9  0.0  7.7  1.9  88.5 |

**Table 4b** Frequency of the use of supraglottic airway device in the past year per age category per specialty in percentages.

*Ped = Pediatricians, Ane = Anesthetists. Neonate 0-4 weeks, infant 4 weeks-1 year, child 1-16 years.*

|  | **Neonate** | | **Infant** | | **Child** | |
| --- | --- | --- | --- | --- | --- | --- |
|  | **Ped** | **Ane** | **Ped** | **Ane** | **Ped** | **Ane** |
| **Never**  **1 time**  **2-5 times**  **6-10 times**  **>10 times** | 98.1  0.0  1.9  0.0  0.0 | 94.2  3.8  0.0  0.0  1.9 | 100  0.0  0.0  0.0  0.0 | 38.5  9.6  32.7  13.5  5.7 | 100  0.0  0.0  0.0  0.0 | 1.9  0.0  13.5  3.8  80.8 |

**Table 5a** Frequency of endotracheal intubation training in the past year per specialty in percentages.

| \|  \| **Pediatricians** \| **Anesthetists** \| \| --- \| --- \| --- \| \| **Never**  **Once a year**  **Once half a year**  **Once a month** \| 48.1  28.9  17.3  5.7 \| 63.5  9.6  5.7  21.2 \| |
| --- | --- | --- | --- | --- | --- | --- |

**Table 5b** How endotracheal intubation training was performed in the past year per specialty in percentages.

| \|  \|  \| \| --- \| --- \|  \|  \| **Pediatricians** \| **Anesthetists** \| \| --- \| --- \| --- \| \| **On a manikin**  **In operating theatre (in patients)**  **In operating theatre (in patients) and on manikin** \| 93.8  2.1  4.1 \| 23.3  53.4  23.3 \| |
| --- | --- | --- | --- | --- | --- | --- | --- | --- |
