# Supplementary material for: Endotracheal intubation skills of pediatricians versus anesthetists in neonates and children
Source: Eur J Pediatr. 2019 Jun 8;178(8):1219–27. doi: 10.1007/s00431-019-03395-8 (PMC6647518; doi:10.1007/s00431-019-03395-8)
Supplement: Supplementary file 2 — (DOCX 101 kb) [file 431_2019_3395_MOESM2_ESM.docx]

**Appendix A.** Questionnaire

Participant number:

1. What is your specialty?

• Pediatrician

• Anesthetist

2. What is your age?

• < 40 years

• Between 40-50 years

• Between 51-60 years

• > 60 years

3. Time since board-certification in years:

• < 5 years

• Between 5-10 years

• Between 11-20 years

• > 20 years

4. In which city do you work? (open question)

5. In what kind of hospital do you work?

• Large general hospital

• Middle-large general hospital

• Small general hospital

6. Do you have a subspecialty? If yes, which subspecialty?

• Yes, I am ……

• No

7. Are you a consultant pediatrician/anesthetist with on-call responsibilities for pediatric and neonatal care?

• Yes

• No

8.a. How many times in the past year did you perform bag-mask ventilation or did you use a Neopuff (T-piece) on a newborn (<24 hours after birth)?

Never / once / 2-5 times / 6-10 times / > 10 times

8.b. How many times in the past year did you use a supraglottic airway device in a newborn (<24 hours after birth)?

Never / once / 2-5 times / 6-10 times / > 10 times

8.c. How many times in the past year did you perform endotracheal intubation on a newborn (<24 hours after birth)?

Never / once / 2-5 times / 6-10 times / > 10 times

9.a. How many times in the past year did you perform bag-mask ventilation on an infant?

Never / once / 2-5 times / 6-10 times / > 10 times

9.b. How many times in the past year did you use a supraglottic airway device in an infant?

Never / once/ 2-5 times / 6-10 times / > 10 times

9.c. How many times in the past year did you perform endotracheal intubation on an infant?

Never / once / 2-5 times / 6-10 times / > 10 times

10.a. How many times in the past year did you perform bag-mask ventilation on a child?

Never / once / 2-5 times / 6-10 times / > 10 times

10.b. How many times in the past year did you use a supraglottic airway device in a child?

Never / once / 2-5 times / 6-10 times / > 10 times

10.c. How many times in the past year did you perform endotracheal intubation on a child?

Never / once / 2-5 times / 6-10 times / > 10 times

11. Do you find yourself competent to perform an endotracheal intubation on a newborn in an acute setting?

Not at all competent / not competent / neutral / competent / very competent / don’t

know

12. Do you find yourself competent to perform an endotracheal intubation on an infant in an acute setting?

Not at all competent / not competent / neutral / competent / very competent / don’t

know

13. Do you find yourself competent to perform an endotracheal intubation on a child in an acute setting?

Not at all competent / not competent / neutral / competent / very competent / don’t

know

14. How many times do you train the following skills?

a. Neopuff (T-piece) ventilation

Never / once a month / every 6 months / once a year b. Bag-mask ventilation

Never / once a month / every 6 months / once a year c. Laryngeal airway mask insertion

Never / once a month / every 6 months / once a year d. Endotracheal intubation

Never / once a month / every 6 months / once a year

15. How do you train your skills? (multiple answers possible)

• On a manikin

• In the operation room

• On the intensive care unit

• During EPALS / APLS or other courses

• Otherwise…

16. Are there any agreements on which person will intubate a neonate or child in an acute care setting in your hospital?

Yes / No / Don’t know

16a. If yes, who will intubate the neonate or child in an acute care setting?

• Pediatrician

• Anesthetist

• For neonates the pediatrician, for children the anesthetist

• Intensivist

• ER-doctor

• Don’t know

• Otherwise…..

17. Are there any written agreements (protocol) about who will intubate a neonate or

child in an acute care setting in your hospital?

Yes / No / Don’t know

17a. If yes, who will intubate the neonate or child in an acute care setting?

• Pediatrician

• Anesthetist

• For neonates the pediatrician, for children the anesthetist

• Intensivist

• ER-doctor

• Don’t know

• Otherwise…..

18. Is it your opinion that it is preferred that children are intubated by an anesthetist?

Not at all preferred / not preferred / neutral / preferred / very preferred

Explanation (optional):

19. Is it your opinion that it is preferred that neonates are intubated by an anesthetist?

Not at all preferred / not preferred / neutral / preferred / very preferred

Explanation (optional):

# Appendix B. Total performance scoring list

# Total performance scoring list endotracheal intubation of neonatal manikin

| Observer: | Participant: Date: | |
| --- | --- | --- |
| **Choice of materials** |  | |
| Laryngoscope | Miller 1 Mac 1 Mac 2 |  |
| Tube size | tube 2.5 tube 3.0 tube 3.5 tube 4 |  |
| **Intubation (Total performance score)** | **Points** | |
| Pre-oxygenation technique ^1^ | 3 neutral position, adequate CE-grip, frequency 40-60/min  2 minus 1 item  1 minus 2 items  0 minus 3 items  −2 not conducted |  |
| Duration of the successful attempt ^2^ | 1 ≤ 30 seconds  −1 31 sec - 60 sec  −2 ˃ 60 sec |  |
| Duration all attempts (max 3) ^3^ | 3 ≤ 30 sec  2 31 sec - 60 sec  1 61 sec - 120 sec  0 ˃ 120 sec  −1 No successful intubation or ≥ 4 attempts |  |
| Number of attemps ^4^ | 3 1 attempt  2 2 attempts  1 3 attempts  −1 ≥ 4 attempts or no successful intubation |  |
| Bag-mask ventilation between attempts | 2 ventilation between all attempts  −2 no ventilation between one or more attempts |  |
| Cormack-Lehane Classification^5^  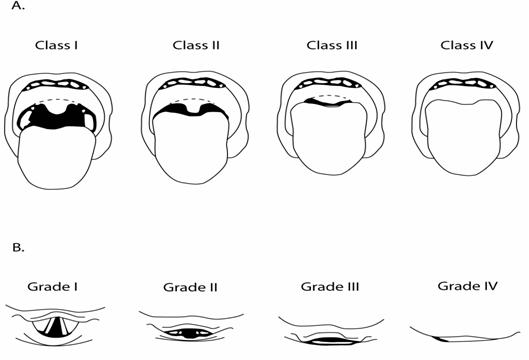 | 2 grade 1: most of the glottis can been seen  1 grade 2: only posterior portion of glottis or only arytenoid  cartilages are visible  0 grade 3: only epiglottis seen  −1 grade 4: neither glottis nor epiglottis seen |  |
| Potential complicating factors ^6^ | −2 laryngoscope blade through vocal cords / causing  deformation |  |
|  | −1 switch laryngoscope to other hand during intubation |  |
|  | −2 tube in oesophagus |  |
|  | −2 incorrect cuff placement: between vocal cords |  |
| Tube position | 1 correct (oral 10-11cm and nasal 11-12cm)  −1 too shallow (oral < 10cm and nasal < 11 cm)  −1 too deep (oral > 11cm and nasal > 12cm) |  |
| **Check tube position** | **points** | |
| Thoracic excursion  Auscultation lung  Auscultation stomach  Laryngoscopy  Saturation  Capnography / End-tidal CO_2_ | 3 ≥ 3 items done  2 2 items done  1 1 item done  −2 no control |  |
| **End-assessment grade** | | |
| 1------2------3-------4-------5-------6--------7--------8-------9-------10 | | |
| € Not sufficiently qualified € Sufficiently qualified | | |

^1^ Pre-oxygenation technique: Head in neutral position, correct holding of mask and mandibula according CE-grip, adequate ventilation frequency (40-60/minute).

^2^ Duration of successful intubation attempt: Duration of successful intubation attempt was defined as the time from introduction of the laryngoscope blade into the mouth to the time it was removed during the successful intubation attempt. (Successful intubation attempt was defined as the tube passing the vocal cords.)

^3^ Duration of all intubation attempts (max 3): was defined as the time from introduction of the laryngoscope blade into the mouth to the time it was removed during all intubation attempts, irrespective of whether a tube was introduced during this attempt. With a maximum of 3 attempts in total.

^4^ Number of attempts: was defined as the number of attempts that is needed for successful intubation.

^5^ Cormack-Lehane classification: was defined as the glottic visibility according to the Cormack-Lehane classification observed during the successful intubation attempt.

^6^ Potential complicating factors: Manoeuvres that can cause traumatic injury or oedema to the vocal cords, mucosa or larynx. Manoeuvres that represent inadequate intubation technique and/or insufficient anatomic knowledge.

Total performance scoring list endotracheal intubation of child manikin

| Observer: | Participant: Date: | |
| --- | --- | --- |
| **Choice of materials** |  | |
| Laryngoscope | Mac 2 Mac 3 Mac 4 |  |
| Tube size | tube 4 tube 4.5 tube 5  tube 5.5 tube 6 tube 6.5 |  |
| **Intubation (Total performance score)** | **Points** | |
| Pre-oxygenation technique ^1^ | 3 sniffing position, adequate CE-grip, frequency 20-40min  2 minus 1 item  1 minus 2 items  0 minus 3 items  −2 not conducted |  |
| Duration of the successful attempt ^2^ | 1 ≤ 30 seconds  −1 31 sec - 60 sec  −2 ˃ 60 sec |  |
| Duration all attempts (max 3) ^3^ | 3 ≤ 30 sec  2 31 sec - 60 sec  1 61 sec - 120 sec  0 ˃ 120 sec  −1 No successful intubation or ≥ 4 attempts |  |
| Number of attemps ^4^ | 3 1 attempt  2 2 attempts  1 3 attempts  −1 ≥ 4 attempts or no successful intubation |  |
| Bag-mask ventilation between attempts | 2 ventilation between all attempts  −2 no ventilation between one or more attempts |  |
| Cormack-Lehane Classification^5^  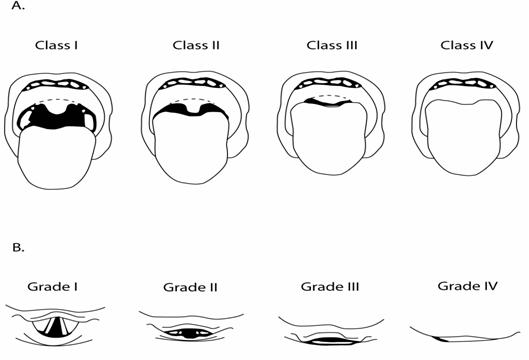 | 2 grade 1: most of the glottis can been seen  1 grade 2: only posterior portion of glottis or only  arytenoid cartilages are visible  0 grade 3: only epiglottis seen  −1 grade 4: neither glottis nor epiglottis seen |  |
| Potential complicating factors ^6^ | −2 laryngoscope blade through vocal cords / causing  deformation |  |
|  | −1 switch laryngoscope to other hand during intubation |  |
|  | −2 tube in oesophagus |  |
|  | −2 incorrect cuff placement: between vocal cords |  |
| Tube position | 1 correct (oral 15-17cm and nasal 20-21cm)  −1 too shallow (oral <15cm and nasal < 20 cm)  −1 too deep (oral >17cm and nasal > 21cm) |  |
| **Check tube position** | **Points** | |
| Thoracic excursion  Auscultation lung  Auscultation stomach  Laryngoscopy  Saturation  Capnography / End-tidal CO_2_ | 3 ≥ 3 items done  2 2 items done  1 1 item done  −2 no control |  |
| **End-assessment grade** | | |
| 1------2------3-------4-------5-------6--------7--------8-------9-------10 | | |
| € Not sufficiently qualified € Sufficiently qualified | | |

^1^ Pre-oxygenation technique: Head in sniffing position, correct holding of mask and mandibula according CE-grip, adequate ventilation frequency (20-40/minute).

^2^ Duration of successful intubation attempt: Duration of successful intubation attempt was defined as the time from introduction of the laryngoscope blade into the mouth to the time it was removed during the successful intubation attempt. (Successful intubation attempt was defined as the tube passing the vocal cords.)

^3^ Duration of all intubation attempts (max 3): was defined as the time from introduction of the laryngoscope blade into the mouth to the time it was removed during all intubation attempts, irrespective of whether a tube was introduced during this attempt. With a maximum of 3 attempts in total.

^4^ Number of attempts: was defined as the number of attempts that is needed for successful intubation.

^5^ Cormack-Lehane classification: was defined as the glottic visibility according to the Cormack-Lehane classification observed during the successful intubation attempt.

^6^ Potential complicating factors: Manoeuvres that can cause traumatic injury or oedema to the vocal cords, mucosa or larynx. Manoeuvres that represent inadequate intubation technique and/or insufficient anatomic knowledge.
